# Acquired Hepatocerebral Degeneration in a Septic Patient: Hospital Course and MRI Findings

**DOI:** 10.7759/cureus.78511

**Published:** 2025-02-04

**Authors:** Ethan L Porter, Alexandria R Koney, Alexia R Jauregui, Emelie N McQuitty, Courtney T Huynh, Rita B Khouri, Alisha Kashyap, Matthew T Wong

**Affiliations:** 1 Internal Medicine, John P. and Kathrine G. McGovern Medical School, University of Texas Health Science Center-Houston, Houston, USA

**Keywords:** acquired hepatocerebral degeneration, alcohol abuse, alcoholic cirrhosis, globus pallidus, hyperammonemia, mri finding, septic shock

## Abstract

Acquired hepatocerebral degeneration (AHD) is a rare neuropsychiatric disorder affecting cirrhotic patients, often presenting with neuropsychiatric symptoms triggered by a precipitating factor. Magnetic resonance imaging (MRI) showing T1 hyperintensities in the globus pallidus is diagnostic. In this case report, a 40-year-old female patient with alcoholic cirrhosis presented with generalized swelling, jaundice, fever, and signs of septic shock. After stabilization, changes in cognition while this patient was hospitalized prompted the management of several different factors, including the underlying infection, hypernatremia, vitamin deficiency, alcohol withdrawal, and hyperammonemia. Infectious disease consultation helped determine the antibiotic selection and dosage for bacteremia and later concerns for meningitis. This case report illustrates the role of MRI in diagnosing AHD and highlights the importance of how managing precipitating factors such as infection and hyperammonemia is key to improving cognitive status.

## Introduction

Acquired hepatocerebral degeneration (AHD) is a rare, chronic condition affecting about 1% of individuals with liver cirrhosis [1]. Its clinical manifestation is neurological, with a characteristic tremor as well as altered mental status (AMS), and it is officially diagnosed with a characteristic magnetic resonance imaging (MRI) finding: a T1-weighted hyperintensity in the globus pallidus [2]. The goal of treatment during exacerbations of hepatic encephalopathy in patients with this condition focuses primarily on managing ammonia levels and correcting the underlying precipitating factor, including sepsis or volume depletion. This case report will focus on a patient who initially presented with septic shock from multiple underlying infections with an MRI finding of AHD observed later in her hospital course and how this patient was stabilized and managed during hospitalization.

## Case presentation

The patient, a woman with a history of alcohol use disorder and alcoholic cirrhosis, presented to the emergency room with generalized swelling, scleral icterus and jaundice, petechiae, bruising, and weakness. She also complained of subjective fever, chills, myalgia, and arthralgia. Vital signs demonstrated hypotension and hypothermia. Initial laboratory assessment results were concerning for several abnormalities, including anemia, with a hemoglobin concentration of 7.3 g/dL (reference range: 12.0 g/dL to 16.0 g/dL); thrombocytopenia, with a platelet count of 23,000/uL (reference range: 150,000/uL to 400,000/uL); lactic acidosis, with a lactic acid concentration of 9.4 mmol/L (reference range: 0.5 mmol/L to 2 mmol/L) and an arterial blood gas showing a pH of 7.24 (reference range: 7.35 to 7.45) and partial pressure of CO_2_ (pCO_2_) of 29.4 mm Hg (reference range: 32.0 mm Hg to 45.0 mm Hg); acute kidney injury, with a creatinine concentration of 5.6 mg/dL (reference range: 0.6 mg/dL to 1.2 mg/dL); and hypoglycemia, with a blood glucose concentration of 32 mg/dL (reference range: 70 mg/dL to 110 mg/dL). Additionally, a urinalysis was positive for nitrates (reference is negative) and showed 49 white blood cells per high power field (reference range: 0 to 5 white blood cells per high power field), which was concerning for a urinary tract infection. These findings prompted urine cultures, which grew extended-spectrum beta-lactamase (ESBL) *E. coli*. She was diagnosed with septic shock, decompensated alcoholic cirrhosis, and acute kidney injury and was transferred to the Medical Intensive Care Unit (MICU) for stabilization.

In MICU, because of septic shock due to *E. coli*, the patient was intubated and treated with broad-spectrum antibiotics meropenem and vancomycin, pressors, and fluids, which improved her blood pressure and urine output. Shortly thereafter, on day two of hospitalization, the medicine team reassessed the patient with a brief neurological exam; the patient was only alert and oriented only to self (A&Ox1), compared to her reported baseline of alert and oriented to person, place, and time (A&Ox3). According to the Clinical Institute Withdrawal Assessment (CIWA) protocol, benzodiazepines were administered for alcohol withdrawal. Her ammonia concentration was recorded at 78 umol/L (reference range: 16.0 umol/L to 53.0 umol/L), and she received thiamine, vitamins, electrolytes (magnesium, potassium, and phosphate), and lactulose titrating up to 3-4 bowel movements per day, which helped lower the ammonia concentration to 40 umol/L, which is within the reference range. Despite these interventions, her mental status remained fluctuant between A&Ox1 and A&Ox3 in MICU.

Blood cultures grew *Neisseria gonorrhoeae*, prompting infectious disease consultation. Wrist arthralgia was noted, with imaging proving negative for trauma and the surgical team not being concerned about septic arthritis. The differential for her AMS included infection, hyponatremia, vitamin deficiency, and hyperammonemia. She continued the treatments initiated in the MICU and was switched to ceftriaxone for *Neisseria gonorrhoeae* bacteremia on day six of hospitalization.

On day 11 of hospitalization, the patient developed neck rigidity to flexion, a non-blanchable purpuric rash on the upper extremities, and deteriorating mental status to A&Ox1. Meningitis was suspected, and a lumbar puncture (LP) was scheduled; however, attempts at an LP proved initially unsuccessful due to body habitus, so interventional radiology was consulted to perform the LP at a later time. In the interim, a computed tomography (CT) scan found ventricular enlargement but could not determine meningitis, prompting an MRI of the brain. She was started on a broad antibiotic regimen (ampicillin, vancomycin, ceftriaxone, and acyclovir) for potential meningitis.

**Figure 1:**
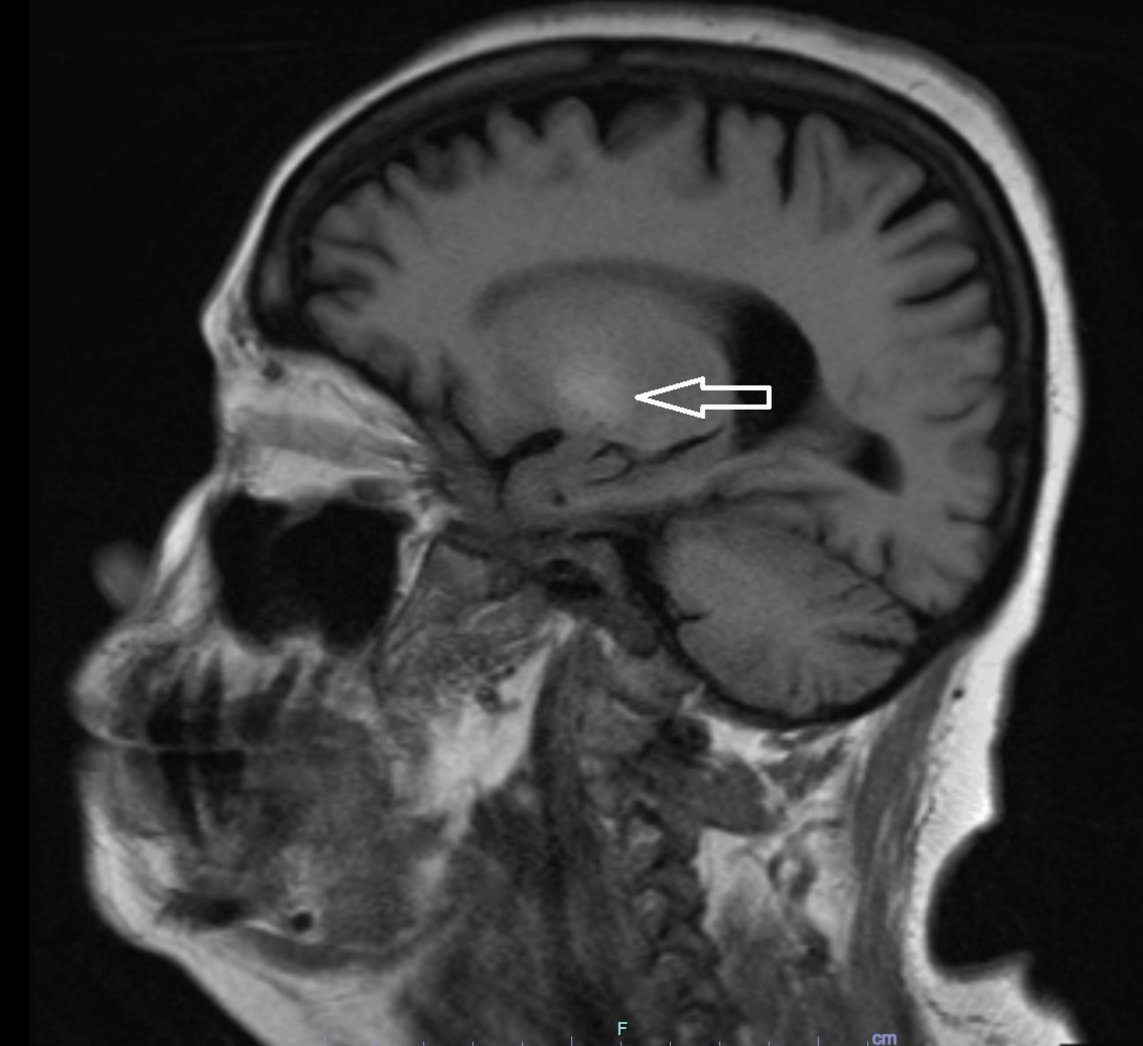
Sagittal T1-weighted MRI of the brain, with the arrow marking the globus pallidus hyperintensity

**Figure 2:**
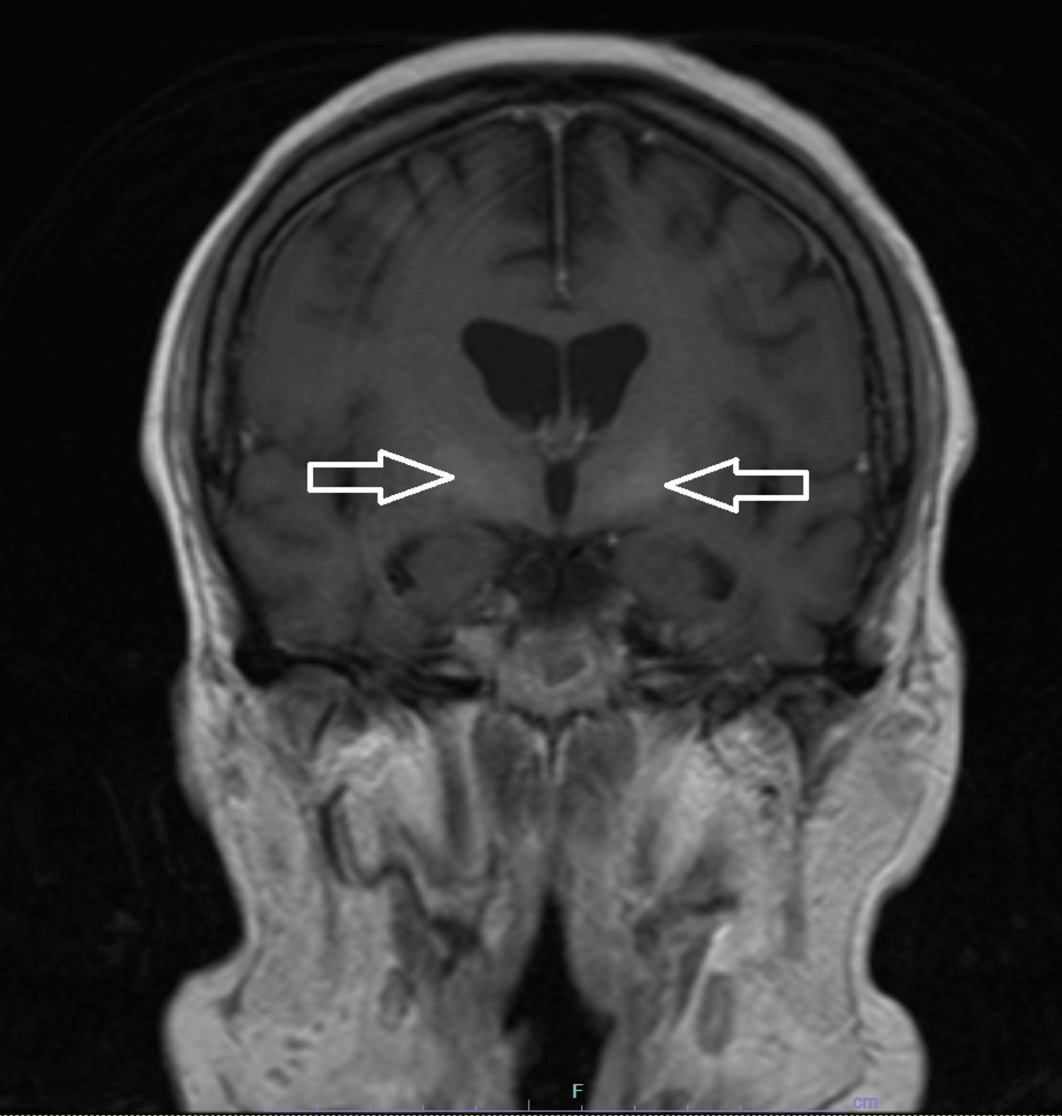
Coronal T1-weighted MRI of the brain, with the arrows marking the globus pallidus hyperintensity bilaterally

**Figure 3:**
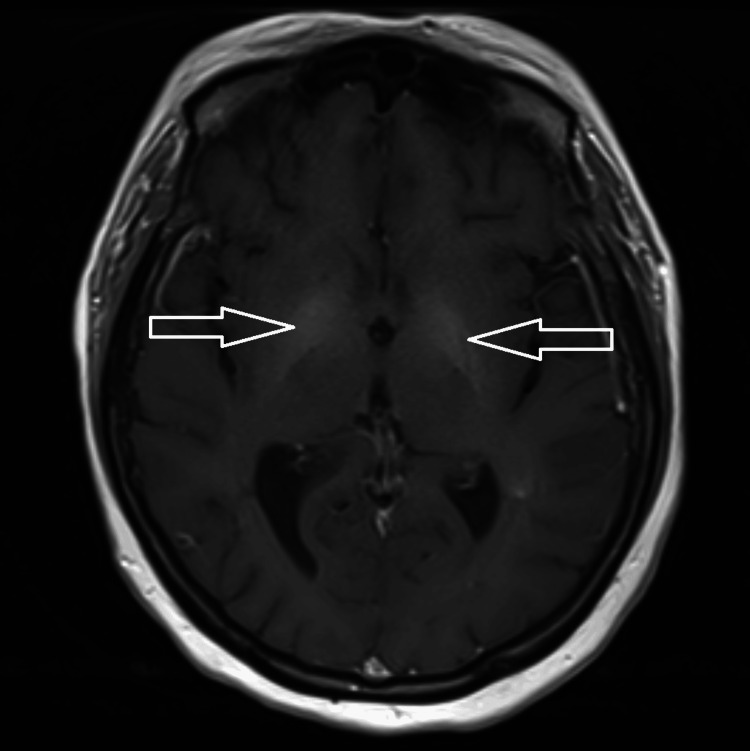
Axial T1-weighted MRI of the brain, with the arrows pointing to the globus pallidus hyperintensity bilaterally

The brain MRI showed mild cerebral volume loss and hyperintense T1 signals in the globus pallidus. As follows are the brain MRI T1-weighted sagittal (Figure 1), coronal (Figure 2), and axial (Figure 3) views, with the hyperintensity in the globus pallidus region of the brain clearly marked in each image.

On day 16 of hospitalization, LP cultures were negative, removing suspicion of meningitis. The patient tested positive for Herpes Simplex Virus 1 (HSV-1) from a genital lesion swab. By day 19, she had received 14 days of ceftriaxone for the treatment of *Neisseria gonorrhoeae*, and on day 23, she was discharged with plans for continued valacyclovir treatment for HSV-1. Unfortunately, the patient succumbed to sepsis-related complications a week postdischarge at a different hospital.

## Discussion

Hepatocerebral degeneration was so named to describe the changes in neurological function which occur after long-standing liver failure. There are two major forms: familial, related to Wilson’s disease and other inherited conditions, and acquired, the vast majority of which are related to alcoholic cirrhosis [3].

It is important to note the difference between these two forms because the two conditions are treated differently. Clinically, the difference between the two is typically the age of onset and the presence of other symptoms. Wilson’s disease typically manifests in childhood or young adulthood, whereas the acquired form progresses gradually and most often becomes apparent later in life, although occasionally it has an earlier onset [4]. Additionally, Wilson’s disease is associated with other clinical symptoms such as Kayser-Fleischer rings and arthralgia, which may be absent in the acquired form [5]. These differences can be used to differentiate Wilson’s disease from AHD. This patient did have wrist arthralgia later during her admission; however, considering the patient had a late onset of symptoms and lacked other symptoms of Wilson’s disease on presentation, as well as a confirmed history of alcohol use disorder, Wilson’s disease was not on the differential for the patient. Instead, the cirrhosis and resulting hepatic encephalopathy and hepatocerebral degeneration were more likely due to chronic alcohol use.

Neuropsychiatric changes are associated with AHD, which include tremors and AMS, such that a patient may demonstrate unawareness of their person, place, time, or situation [3]. These changes are known to resolve temporarily [3]. These changes are associated with a number of underlying mechanisms. First, generally in severe hepatic injury, the liver is unable to metabolize ammonia into urea for proper excretion, leading to increased depositing of ammonia in the brain in conjunction with a portosystemic shunt [6]. Second, end-stage liver disease can cause prolonged states of neuroinflammation, changes in neuronal transmission, and alterations in blood-brain barrier permeability [7]. Finally, specific to AHD, the described “degeneration” is widespread in the brain, as pathologic abnormalities are found in the glial cells and neurons in the cortex. However, the degeneration is localized to the basal ganglia as well, contributing to a wide variety of movement disorders [8].

The most significant MRI finding for AHD is the signal hyperintensity on T1-weighted imaging in the internal pallidum of the globus pallidus [2]. Although the globus pallidus hyperintensity is the most common, a majority of patients can have these hyperintensities elsewhere; up to 75% of patients with AHD have extrapallidal involvement [1]. These hyperintensities outside the globus pallidum include the caudate nucleus, putamen, internal capsule, mesencephalon, cerebellum, and periaqueductal gray matter [2,9]. T1-weighted imaging highlights anatomical structures by differentiating cerebrospinal fluid, gray matter, and white matter, aiding in the detection of abnormalities [10]. In this patient, the hyperintensity in the globus pallidus region of the brain can be seen clearly from all three views: sagittal, axial, and coronal.

This patient’s wide differential for AMS and how she was treated during her hospitalization was important because the treatment of hepatic encephalopathy in general focuses on controlling two factors: ammonia levels and precipitating factors [11]. Since AHD is a chronic and recurring form of hepatic encephalopathy, it is important to note that the most important long-term treatment to prevent the worsening of AHD and therefore prevent the worsening of the aforementioned mental status and neurologic changes is a liver transplant [11]. First, to reduce ammonia, lactulose, which is a nonabsorbable disaccharide, is often used to acidify the gut and thereby reduce the ammonia produced by the gut [12]. This was titrated in our patient to about 3-4 bowel movements per day. Another medication that is often used to achieve the same purpose in lowering ammonia levels is rifaximin, which also suppresses ammonia production in the gut [12]. Second, precipitating factors for hepatic encephalopathy include sepsis, dehydration, hyponatremia, use of sedatives or narcotics, and many others, any and all of which should be treated accordingly and eliminated [12]. This patient’s sepsis was treated with the appropriate regimen of fluids, pressors, and antibiotics. This patient was also treated by correcting electrolyte abnormalities, supplementing the patient with thiamine, and offering a benzodiazepine conservatively when the patient was at risk for alcohol withdrawal symptoms. Therefore, by covering many different precipitating factors, it was possible to address some triggers for the tremor and the AMS.

## Conclusions

This case of a female in her 40s with a history of alcoholic cirrhosis and severe alcohol use disorder illustrates the complexities of managing AHD amid severe liver dysfunction and infection. The patient's deteriorating mental status required a broad differential, including hyperammonemia, infectious causes, electrolyte derangement, vitamin deficiencies, and alcohol withdrawal. The treatment approach combined lactulose administration, antibiotics, electrolyte correction, thiamine supplementation, and conservative usage of benzodiazepines, which helped address this form of hepatic encephalopathy. The MRI findings, including hyperintensity in the globus pallidus, demonstrate structural changes. Finally, early collaboration with the infectious disease team was crucial in the management of both bacteremia and potential meningitis. Overall, this case included a thorough initial assessment, radiologic diagnosis, and hospitalist management of a patient with AHD, ensuring that both hepatic and neurological complications were addressed to optimize the hospitalized patient.
